# Changes in soil bacterial community triggered by drought-induced gap succession preceded changes in soil C stocks and quality

**DOI:** 10.1002/ece3.409

**Published:** 2012-11-02

**Authors:** Jorge Curiel Yuste, Josep Barba, Antonio José Fernandez-Gonzalez, Manuel Fernandez-Lopez, Stefania Mattana, Jordi Martinez-Vilalta, Pau Nolis, Francisco Lloret

**Affiliations:** 1 Museo Nacional de Ciencias Naturales (MNCN), CSICSerrano 115 dpdo, E-28006, Madrid, Spain; 2 Centre de Recerca Ecológica i Aplicacions Forestals (CREAF); Edifici C, Universitat Autònoma de BarcelonaE-08193, Bellaterra, Barcelona, Spain; 3 Servei de Ressonància Magnètica Nuclear, Universitat Autònoma de Barcelona08193, Bellaterra, Spain; 4 Estacion Experimental del Zaidin (EEZ), CSIC, Profesor Albareda, 1E-18008, Granada, Spain

**Keywords:** Climate change, drought, ecosystem functioning, forest dieback, gap colonization, microbial diversity, nutrient cycling, pyrosequencing, tree mortality

## Abstract

The aim of this study was to understand how drought-induced tree mortality and subsequent secondary succession would affect soil bacterial taxonomic composition as well as soil organic matter (SOM) quantity and quality in a mixed Mediterranean forest where the Scots pine (*Pinus sylvestris*) population, affected by climatic drought-induced die-off, is being replaced by Holm-oaks (HO; *Quercus ilex*). We apply a high throughput DNA pyrosequencing technique and ^13^C solid-state Nuclear Magnetic Resonance (CP-MAS ^13^C NMR) to soils within areas of influence (defined as an surface with 2-m radius around the trunk) of different trees: healthy and affected (defoliated) pines, pines that died a decade ago and healthy HOs. Soil respiration was also measured in the same spots during a spring campaign using a static close-chamber method (soda lime). A decade after death, and before aerial colonization by the more competitive HOs have even taken place, we could not find changes in soil C pools (quantity and/or quality) associated with tree mortality and secondary succession. Unlike C pools, bacterial diversity and community structure were strongly determined by tree mortality. Convergence between the most abundant taxa of soil bacterial communities under dead pines and colonizer trees (HOs) further suggests that physical gap colonization was occurring below-ground before above-ground colonization was taken place. Significantly higher soil respiration rates under dead trees, together with higher bacterial diversity and anomalously high representation of bacteria commonly associated with copiotrophic environments (r-strategic bacteria) further gives indications of how drought-induced tree mortality and secondary succession were influencing the structure of microbial communities and the metabolic activity of soils.

## Introduction

The increase in drought-induced mortality and forest decline that has been recorded around the globe during the last decades suggests that some of the world's forest ecosystems are already responding to climate change (Van Mantgem et al. 2009; Allen et al. 2010; Martínez-Vilalta et al. 2011). This die-off is characterized by rapid defoliation and progressive increase in the mortality of over-story trees (Breda et al. 2006). Such widespread mortality events have the capacity to transform regional landscapes on a subdecadal timescale, with significant implications for stand structure and dynamics (Royer et al. 2011; McDowell 2011). In this context, ecosystems of the Mediterranean areas of the world are very vulnerable to current climate change (Carnicer et al. 2011) and large-scale predictions for the Mediterranean basin suggest that areas currently occupied by cold-adapted species will be replaced by species better adapted to drought (Ruiz-Labourdette et al. 2012; Serra-Diaz et al. 2012).

While there already exists a critical mass of information available about the location of mortality events around the world and of the symptoms and causes of tree mortality (see Allen et al. 2010 for a review), the implications of tree die-off and subsequent succesional processes on ecology of soil microbial communities and soil biochemistry has remained surprisingly unexplored. Because soil feedbacks are predicted to exert a strong, although still rather uncertain, impact on climate (Meir et al. 2006; Heimann and Reichstein 2008), by substantially accelerating or decelerating soil CO_2_ emissions (Cox et al. 2000; Friedlingstein et al. 2006), it is important to study mechanisms by which forest decline could possibly affect soil C and nutrients cycling. In particular, it is crucial to understand how forest decline may affect the ecology of soil C pools and microbial communities, which comprise a large portion of the genetic diversity on Earth (Whitman et al. 1998) and are responsible for the oxidation of soil C (Aerts 2006; Balser and Wixon 2009; Curiel Yuste et al. 2011).

There exists an extensive literature on the strong dependency of soil metabolic activity on plant activity, which is a multiscalar issue (e.g., Högberg et al. 2001; Janssens et al. 2001, Bahn et al. 2008, Vargas et al. 2010). Plants provide microbes with organic carbon (C) forms of very different nature and palatability, from organic C forms of very fast turnover times (e.g., exudates) to organic C forms of very low palatability and slow turnover times (e.g., dead wood). However, plant activity may also influence microbial-mediated soil organic matter (SOM) decomposition in other ways, for example, stimulating SOM decomposition by exudates supply (e.g., Fontaine et al. 2007). Moreover, the chemistry of plant litter, which strongly affect the physicochemical properties of soil (Binkley 1995), exert also a strong effect over the composition and structure of soil microbial communities (Kuiters 1990; Kraus et al. 2003; Strickland et al. 2009; Ushio et al. 2010). It is therefore likely that the eco-physiological and ecological processes associated with drought-induced forest die-off and secondary succession will affect both, the ecology and functioning of soil microbial communities, although we do not know at which extent.

The limitations associated with traditional methodologies to describe the enormous soil microbial diversity has been a barrier to studying their ecology (Fierer et al. 2007). However, large-scale pyrosequencing of partial 16S rRNA genes has recently emerged as a powerful tool, as it provides a vast number of sequences that can be compared with clone libraries and assigned to an updated taxonomical status (Petrosino et al. 2009). In this context, a number of recent studies have discovered a vast, unprecedented microbial diversity in soils and a large heterogeneity of microbial communities across biomes (Roesch et al. 2007; Jones et al. 2009; Lauber et al. 2009), land uses (Acosta-Martínez et al. 2008; Will et al. 2010; Nacke et al. 2011), and soil horizons (Will et al. 2010), which far exceeded the values obtained using the commonly used fingerprinting techniques, such as terminal restriction fragment length polymorphisms (see Curiel Yuste et al. 2011 for an example).

Recent applications of solid-state cross-polarization magic angle spinning (CP MAS) ^13^C nuclear magnetic resonance (NMR) spectroscopy to soils have also increased our understanding of the chemical structure of SOM (Golchin et al. 1995; Kögel-Knabner 1997, 2000 and Preston 2001). ^13^C NMR provides a first approximation of the relative abundance of organic C functional groups, which can be used as indicators for different compounds. The relative distributions of these broad C categories in SOM vary with decay and microbial processing (Baldock et al. 1992 and Golchin et al. 1994). Further information on specific types of compounds can be gained from ^13^C NMR spectra by applying a molecular mixing model (Baldock et al. 2004). These techniques, coupled with other ecological analyses, such as microbial ecology and functioning analyses, can provide a powerful assessment of the nature and turnover of soil C pools. Considering many studies have found SOM is a strong driver of community composition (Binkley 1995; Strickland et al. 2009; Ushio et al. 2010), fewer studies have combined in the same study methods for thoroughly characterizing both SOM and microbial community composition.

Here we use an interdisciplinary approach to study how climate-change induced mortality and secondary succession may affect soil C pools and soil respiration as well as the taxonomic composition and diversity of microbial communities. We particularly propose an experimental design consisting in a natural chronosequence of the different stages of Scots pine tree die-off (healthy, defoliated and dead) and of a community of unaffected coexisting HO trees, which are likely to replace Scots pines (successional chronosequence). We performed the study in a forest sited in the Mediterranean basin, a region that may be particularly vulnerable to drought-induced forest die-off (Peñuelas et al. 2001; Martínez-Vilalta and Piñol 2002). We hypothesize that: (1) Tree defoliation and mortality will affect SOM, its quality and/or quality; (2) Even at this local scale, different plant species present a degree of specificity in their relationships with the soil bacterial community; (3) Tree defoliation and mortality will exert a strong influence on the diversity and structure of soil bacterial communities.

## Materials and Methods

### Study site

The study was carried out in a mixed forest in the Titllar Valley in the Prades Mountains (NE Spain: 41°13′N, 0°55′E), between 1010 and 1033 m above sea level (a.s.l.) The climate is typically Mediterranean with a mean annual temperature of 11.2°C, a mean annual rainfall of 720-mm (Climatic Digital Atlas of Catalonia, Ninyerola et al. 2000) concentrated in autumn and spring and a warm and dry summer. The soils are xerochrepts with clay loam texture. However, they are only present in 56% of the study area, whereas the rest of the surface is covered by schist outcrops. Organic horizons cover most of the soil surface with variable thickness. The ground surface of these plots is very unstable, with many superficial mass movements as a consequence of the abundance of stones on the steep slopes (33° on average). Additional information about the study area can be found in Hereter and Sánchez (1999). The mixed forest is basically composed of Scots pine (*Pinus sylvestris*; 45% of the canopy cover and mean diameter at breast height [dbh] of 0.32 m) and the evergreen HO (*Quercus ilex*; 45% of the canopy cover and mean dbh of 0.15 m). The other 10% includes *Quercus cerrioides, Taxus baccata, Prunus mahaleb, Ilex aquifolium, Amelancher ovalis, Sorbus aria, Sorbus torminalis*, and *Cistus laurifolius*. The deciduous *Quercus cerrioides, Sorbus aria*, and *Sorbus torminalis* were sprouting during the sampling period.

**Figure 1:**
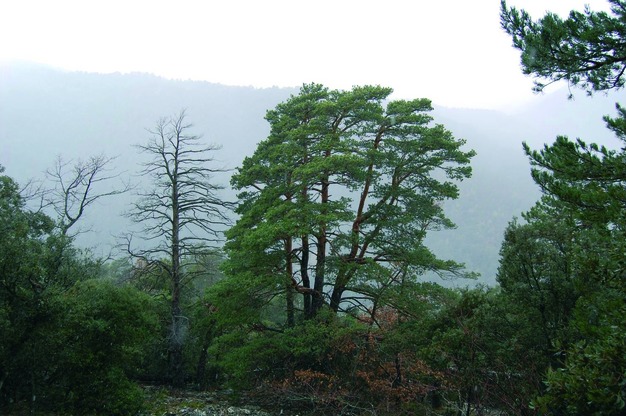
Dead Scots Pine (*Pinus sylvestris*) individual in the mixed forest of the Titllar Valley in Prades (North-East Spain). Photo reproduced by permission of David Aguade.

The Scots pine population is affected by drought-induced die-off, with standing mortality of approximately 20% and varying degrees of defoliation (see Fig. 1) in the surviving trees (Martínez-Vilalta and Piñol 2002; A. Vilà, unpubl. data). The die-off has been documented since 1994 and it has probably been enhanced by the drought episodes occurring in 1998, 2003, and 2005, resulting in recent loss of green canopy and tree mortality (HereŞ et al. 2011). The HO population, however, does not present any visual signs of die-off.

### Experimental set up

The experimental layout consisted of two plots (hereafter called A and B) of about 260 m^2^ (16.2 × 16.2 m) with 100 measurement points distributed in a regular grid within each plot (two consecutive points separated by 1.8 m), resulting in a total of 200 measurement points. The distance between the two plots was <100 m. Both plots were similar in slope, stoniness and stand structure. All samples were collected during spring 2010, during the wet period of the year. Therefore, soils used for these analyses were no water-limited.

Because we were interested in the relationships between plants and microbes, we considered the area of influence of a tree as the area within a 2-meter radius around a given tree, which is well within the area of a tree's influence observed in several studies (Dickie and Reich 2005; Tang and Baldocchi 2005). According to this criterion, we distinguished sampling points as corresponding to one of the four following predefined tree areas of influence, from which we analyzed its respective microbioma: Healthy pine (HP), Defoliated pine (DFP), Dead pine (DP), and HO. This setup provides a natural chronosequence of the stages of pine decline (health, defoliation, death), which are well documented as being caused by climate and tree characteristics (Galiano et al. 2010; Galiano et al. 2011). Pine green canopy was recorded as the indicator of pine defoliation: a pine was considered defoliated when green canopy amounted to <50% of the potential amount of green needles that could be present under the regular climate conditions in the area (Galiano et al. 2011). Then, the following soil samples, out of the total of 200, were selected for analysis: 18 corresponding to the area of influence of HOs, 11 to that of DFPs, 10 to that of DPs and 9 to that of HP. The remaining samples were discarded for this study because they were not unequivocally sited below one of these tree categories.

**Figure 2:**
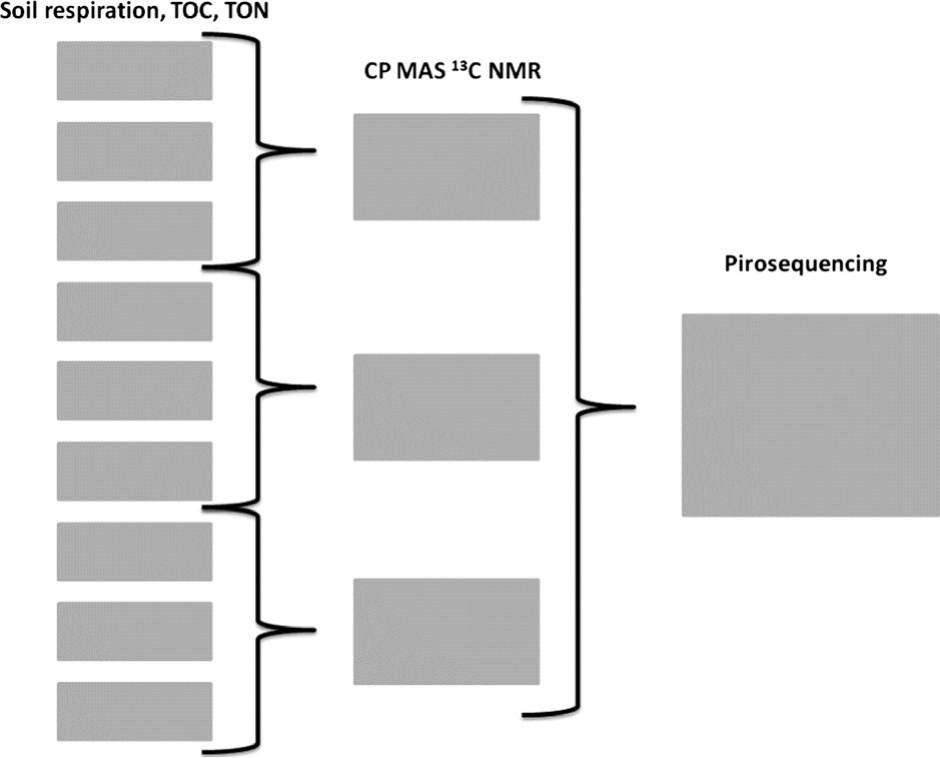
Schematic representation of the experimental design. Some measures (e.g. SR, C and N content; see left small panels of the figure) were performed over each individual soil sample. Nuclear magnetic resonance spectral analyses (central panels) were performed on four samples, previously combined according to proximity (same area of influence or nearest). Pyrosequencing (right panel) was performed on one composite sample, including the equimolar combination of the amplified (PCR) product of each individual samples.

The analysis design for these samples was as follow (see Fig. 2). Analytical variables (soil respiration, C and N content, fine root biomass, C:N of fine roots, pH) were measured at every single sample. For ^13^C NMR, samples were grinded, and then pooled and thoroughly mixed in composite samples (to a total of four per HP, DFP, DP, HO treatment), following the criteria of “area of influence” (samples sharing the same tree area of influence) and/or physical proximity (samples located on areas of influence close to each other; see Fig. 2). For the pyrosequencing analyses, DNA was extracted and amplified (PCR; see below) from each individual soil sample and then mixed in a unique composite sample per treatment (Fig. 2). This was a way to capture the whole diversity of each treatment with a single massive pyrosequencing, with the drawback that variability among soil samples could not be recorded.

### Soil sampling and ancillary data

In spring 2010, soil respiration was measured in the whole grids before soil sampling twice in two consecutive days, using the soda lime technique (Keith and Wong 2006). Soda lime is a mixture of sodium and calcium hydroxides that reacts with CO_2_ to form carbonates. The differences in weight after exposure to a biological source of CO_2_ in a closed system can be converted to respiration rates. This technique has been widely used in the past, primarily to study the spatial variability in soil respiration (SR) (e.g. Janssens et al. 2000; Pumpanen et al. 2004).

Soil temperature and moisture were also recorded during the sampling periods. Soil temperature was recorded instantaneously at each point, at 4-cm depth, five times per day using a thermometer (HH806U, Multilogger Thermometer; Omega, Stamford, Connecticut). The five measurements were then integrated to obtain the average daily temperature at each single point of the grid. Soil moisture was determined gravimetrically in the laboratory as the difference between fresh and dry weight divided by the dry weight.

Soil samples of the organic horizons were obtained from 200 cores 10.4 cm in diameter. All samples were collected during April and May 2010. Stones were removed in the laboratory and samples were sieved with a mesh of 2 mm. The amount of matter in organic horizons (Litter-O_L_, fermentation-O_F_ and Humic-O_H_) in dry weight was considered the organic matter content. The carbon (C) and Nitrogen (N) content of soil samples, as well as the fine root biomass (<2 mm in diameter), was also measured at each individual sampling point. All this measures were performed on each individual sample (Fig. 2).

Data on the chemical composition of the HO leaf and Scots pine needle litter for the region in which the plots are located were obtained from the Ecological and Forestry Catalan Inventory (http://www.creaf.uab.es/iefc/).

Tree coordinates and diameter at breast height (dbh) of both living and dead trees were recorded at the two plots. Coordinates and dbh were also measured for trees located within a 2-m buffer belt around the plots, to account for edge effects in the spatial and statistical analyses. Then, we calculated the Hegyi competition index (Hegyi 1974) between trees, considering pines (at different stage of defoliation) and HOs indistinctly.

### Solid state magic angle spinning nuclear magnetic resonance

We used Solid state MAS NMR to study differences in SOM composition associated with different areas of influence. Solid state MAS NMR spectra were obtained from soil samples previously mixed (see Fig. 2) using an Oxford wide bore 9.4 T magnet equipped with a Bruker Avance II console (Bruker BioSpin GmbH, Rheinstetten, Germany) and employing a 4-mm H/X-CP MAS probe. Samples were manually powdered and packed into 4-mm ZrO_2_ rotors and sealed with tight fitting Kel-F caps. Sample spinning was performed using dry nitrogen gas. Sample MAS frequency was set to 12 KHz in all experiments. For all samples, ^1^H-^13^C CP experiments were acquired using a CP mixing time of 2 ms. A strong ^1^H decoupling during acquisition time was applied using the two-pulse phase modulation (TPPM) scheme. Spectra were acquired at 20°C temperature controlled using a Bruker BCU unit. For ^13^C experiments, spectral frequency was 100.577 MHz and the NMR chemical shifts are externally referenced to adamantane (major peak positioned at 38.6 ppm).

We used TOPSPIN 2.5 (Bruker BioSpin, Rheinstetten, Germany) to integrate peak areas under the following seven chemical shift regions (and the general C types they represent): 0–45 (alkyl); 45–65 (methoxyl); 65–95 (*O*-alkyl); 95–110 (di-*O*-Alkyl); 110–145 (Aromatic C); 145–165 (phenolic C), and 165–220 ppm (Carboxylic and carbonyl C; Preston et al. 2001; Baldock et al. 2004). Three different SOM quality indexes were created from these data: The aliphaticity index (A/O-a index) was determined as the ratio of the alkyl region divided by the *O*-alkyl region (Baldock et al. 1992). A higher ratio means a greater contribution of alkyl C, such as lipids and other aliphatic compounds. A lower ratio indicates a greater contribution of *O*-alkyl C, including cellulose and hemicelluloses, which are considered more labile than alkyl C to microbial decomposers (Baldock et al. 1992). The recalcitrance index was determined as (alkyl + aromatic C)/(*O*-alkyl + carbonyl and carboxyl C). Aromatic compounds include polyphenols, tannins, and lignin phenols, whereas the carbonyl and carboxyl NMR region represent compounds, such as peptides. Finally, the aromaticity index was determined as the percentage of the Aromatic C (110–160 ppm) to the total signal (0–160 ppm). Aromaticity has been used to characterize the extent of humification of SOM, under the assumption that SOM becomes aromatic during decomposition (Dai et al. 2001).

An “a priori” test of signal detection power of ^13^C CP/MAS NMR methodology was performed by calculating the detection limit and the quantification limit of the obtained signals:


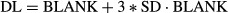



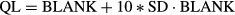


where DL was the “detection limit” of ^13^C CP/MAS NMR, QL was the “quantification limit,” BLANK was the blank sample, SD was the standard deviation of the averaged blank obtained signal.

According to calculations over the raw data, Blank = 0.5 and SD = 0.1. Therefore,


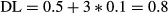



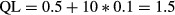


The minimum signal recorded was 6.42 (phenolics in HO), which is eight times bigger than DL and four times bigger than the QL. Therefore, the signals obtained were well within the DLs of this methodology.

### DNA amplification and pyrosequencing

Soil DNA was extracted from each individual soil sample using the PowerSoil™ DNA Isolation Kit (MoBio, Laboratories Inc., CA, USA), following the manufacturer's recommendations. The PowerSoil Isolation Kit differs from the UltraClean Soil DNA isolation kit, as regards its procedure for the removal of humic substance. Briefly, the DNA extraction methods involved chemical lysis of microbial cells with gentle bead-beating. Released DNA was bound to a silica spin filter, which was subsequently washed, and the DNA was recovered in an elution buffer solution. DNA yields and quality were checked after electrophoresis in 0.8% (w/v) agarose gel stained with ethidium bromide under UV light (Sambrook et al. 1989).

Partial bacterial 16S rRNA gene sequences were obtained from the analysis of each individual sample using the coded-primer approach to multiplex pyrosequencing (Binladen et al. 2007). PCR amplification of the hypervariable V4–V5 region of the 16S rRNA gene was performed above each individual soil DNA extraction. PCR was performed using the 8 bp key-tagged universal primers U519F and U926R (Baker et al. 2003). To reduce artifacts associated with PCR, such as chimeras, we adjusted the amplification process following Acinas et al. (2005) modified protocol, consisting on reducing the number of PCR cycles to only 25. We further adjusted the protocol to Roche′s recommendations in order to further diminish artifacts occurrence. The PCR mixtures (25 μL) contained 25 pmol of each primer, 1.8-mmol L^−1^ MgCl_2_, 0.2 mmol L^−1^ dNTPs, 1× the corresponding *Taq* buffer, 1 U of *Taq* Master (5 Prime; Gaithersburg, Maryland) and 10 ng of the same DNA template used above. The PCR program consisted of an initial denaturation step at 94°C for 4 min, 25 cycles of denaturation at 94°C for 15 s, primer annealing at 55°C for 45 s, and extension at 72°C for 1 min, followed by a final step of heating at 72°C for 10 min. For each sample, amplicons were generated in three replicate PCRs. Amplicons of the same treatment (HP, DFP, DP, or HO) were then combined in equimolar amounts in four composite samples (one for each treatment; see Fig. 2 for further information) and subjected to pyrosequencing using the Genome Sequencer Titanium FLX system (454; Life Sciences, Branford, CT, USA) at Life Sequencing S.L. (Valencia, Spain). Sequences were excluded from the analysis following four criteria: (1) If the length read was <150 bp; (2) If the forward primer sequences contained more than two errors; (3) If the sequences had one or more Ns; and, finally; (4) If the quality index, according to the.qual file generated during the pyrosequencing process, was <20. The partial 16S rRNA sequences obtained from each sample are reported as “trimmed” in Table 1. (5) We checked filtered sequences for chimeras using the UCHIME algorithm (Edgar et al. 2011). Results confirmed the low occurrence of chimerical sequences sin our filtered samples (8, 13, 8 and 14 chimeras found in HO, HP, DFP, and DP, respectively).

**Table 1:** Descriptive statistics of the values of the different segment of the spectra and indexes generated (aromaticity, recalcitrancy, and A/A-O ratio) by the ^13^C NMR analyses

						Confidence interval at 95%			
								
		*N*	Average	SD	Standard error	Lower limit	Upper limit	Mín	Máx
Carbonyl C	HO	4	9.13	5.60	2.80	0.22	18.04	2.56	14.22
	HP	3	8.30	2.62	1.51	1.80	14.79	5.34	10.31
	DFP	4	9.93	3.70	1.85	4.04	15.83	4.73	13.43
	DP	4	7.13	4.71	2.36	−0.37	14.63	3.93	14.13
Phenolic C	HO	4	6.42	3.49	1.75	0.86	11.98	2.09	10.06
	HP	3	8.32	2.27	1.31	2.68	13.96	5.96	10.49
	DFP	4	8.92	2.66	1.33	4.69	13.16	5.45	11.81
	DP	4	6.76	4.78	2.39	−0.84	14.36	3.19	13.81
Aryl-C	HO	4	9.81	6.22	3.11	−0.08	19.70	2.75	16.21
	HP	3	11.38	4.50	2.60	0.21	22.55	6.68	15.64
	DFP	4	12.77	3.97	1.98	6.45	19.08	7.84	17.21
	DP	4	9.55	6.34	3.17	−0.54	19.64	4.77	18.85
di-*O*-alkyl	HO	4	8.34	5.19	2.59	0.09	16.60	1.97	13.58
	HP	3	10.15	3.15	1.82	2.32	17.99	6.67	12.82
	DFP	4	11.27	2.81	1.40	6.80	15.74	8.59	14.61
	DP	4	7.53	5.65	2.83	−1.46	16.53	3.16	15.79
*O*-alkyl	HO	4	25.32	14.22	7.11	2.70	47.94	5.01	37.50
	HP	3	28.07	2.65	1.53	21.48	34.66	25.98	31.06
	DFP	4	33.85	6.05	3.03	24.22	43.49	25.30	38.19
	DP	4	23.19	16.00	8.00	−2.27	48.66	9.91	43.84
Metoxyl C	HO	4	9.72	6.22	3.11	−0.17	19.62	2.26	15.41
	HP	3	13.56	9.32	5.38	−9.59	36.70	5.54	23.78
	DFP	4	11.19	3.89	1.94	5.00	17.38	7.11	15.44
	DP	4	7.70	5.21	2.61	−0.60	15.99	4.12	15.41
Alkyl C	HO	4	25.38	13.71	6.85	3.57	47.19	5.01	34.80
	HP	3	30.31	7.93	4.58	10.62	50.00	22.20	38.04
	DFP	4	28.95	3.82	1.91	22.87	35.02	23.82	32.25
	DP	4	22.25	13.87	6.94	0.18	44.33	10.37	37.92
Aromaticity	HO	4	13.28	4.01	2.01	6.90	19.67	9.38	18.44
	HP	3	16.49	2.97	1.72	9.11	23.87	14.40	19.89
	DFP	4	17.13	4.44	2.22	10.06	24.20	13.67	23.52
	DP	4	19.61	5.16	2.58	11.40	27.82	12.81	24.33
Recalcitrancy	HO	4	0.76	0.05	0.02	0.69	0.83	0.70	0.80
	HP	3	0.77	0.07	0.04	0.60	0.95	0.69	0.82
	DFP	4	0.74	0.05	0.03	0.66	0.82	0.68	0.79
	DP	4	0.80	0.03	0.02	0.75	0.85	0.76	0.83
A/A-O ratio	HO	4	0.59	0.12	0.06	0.41	0.78	0.52	0.77
	HP	3	0.60	0.16	0.09	0.20	1.01	0.46	0.78
	DFP	4	0.52	0.04	0.02	0.46	0.57	0.47	0.56
	DP	4	0.59	0.11	0.06	0.41	0.77	0.51	0.74

HP, healthy pine; DFP, defoliated pine; DP, dead pine; H-O, Holm-oaks.

### Taxonomic assignment of sequence reads and diversity indexes

Trimmed sequences were processed through the Ribosomal Database Project (RDP) pyrosequencing pipeline (http://pyro.cme.msu.edu) release 10 (Cole et al. 2009). To avoid bias in the interpretation of the data that could be associated with the different number of samples (*n*) of the four composite samples subjected to pyrosequencing (HP, DFP, DP or HO), all four samples were rarefied to the composite sample with the lowest number of sequences (11,008 Sequences of the HP). Thus, 11,088 sequences were chosen at random from the pool of sequences obtained under the area of influence of the HO, Defoliated, and DP, respectively. The qualified sequences were clustered into operational taxonomic units (OTUs), defined on the basis of a distance of 3%, by complete linkage clustering, and were assigned to phyla by the RDP-II classifier, using an 80% confidence threshold (Wang et al. 2007). Sequences that could not be classified to a phylum at this level of confidence were excluded from subsequent phylum composition analyses.

Each unique sequence was further aligned using the RDP pyrosequencing function aligner to generate phylogenetically ordered rRNA sequences (Nawrocki et al. 2009). Aligned data sets were clustered using the default parameters for the RDP Clustering function. The resulting clusters were used to calculate the Shannon–Weaver (H′) index and the respective evenness, the Chao estimator, and the rarefaction curves (Fig. S1), using the pyrosequencing analysis tools from RDP at the level of 3% dissimilarity, which was considered to approximately correspond with species level.

### Statistical analysis

The RDP sample abundance statistics tool was used to calculate the Jaccard's index to compare the four microbiomes (Chao et al. 2006). Aligned data sets from each microbiome were merged into a single cluster file, which was used to construct a distance matrix at 3% dissimilarity, which produced a dendrogram using the unweighted pair group method with arithmetic mean (UPGMA). We further applied an analysis of molecular variance (AMOVA, mothur, v. 1.21.0) to obtain significant differences between the four communities studied. We applied the default option of 1000 iterations from mothur. This method is widely used in population genetics to test the hypothesis that genetic diversity within two populations is not significantly different from that which would result from pooling the two populations (e.g., Excoffier et al. 1992). Pairwise comparisons were performed using the method of the furthest neighbor.

The four different libraries generated from each tree area of influence were compared pair-wise by estimating the likelihood that the frequency of membership of a given taxon (i.e., phylum, class, order) is the same for the different libraries (in our case, the four microhabitats; Cole et al. 2007, 2009).

We apply a one-way analysis of variance (ANOVA) to further explore significant changes in ecological indicators, such as C content of C:N ratios (Table 2) and differences in ^13^C NMR signals and indexes. One-way ANOVA analyses were complemented using Turkey′s post hoc test to assess differences between treatments. We also explored for significant trends associated with tree die-off using bivariant Pearson correlations between the three different degrees′s of defoliation (healthy, defoliated, and dead) and ecological indicators (e.g., C content, C:N ratios, ^13^C NMR spectral intensity, and indexes).

**Table 2:** Values of different environmental parameters in the four studied microhabitats

	HP	DFP	DP	H-O
Temperature (°C)	14.1 (0.2)	13.9 (0.2)	13.9 (0.1)	13.9 (0.1)
Soil Moisture (g g^−1^)	31.4^a^	59.1^b^	35.6^ab^	30.4^a^
Soil Respiration (mmol m^−2^ s^−1^)	1.8 (0.1)^a^	2.2 (0.1)^ab^	2.7 (0.2)^b^	1.8 (0.1)^a^
Root Biomass (g cm^−2^)	9.7 (1.8)	17.8 (4.7)	18.3 (4.7)	16.0 (2.8)
Root C/N ratio	36.8 (3.8)	31.0 (8.7)	35.7 (5.9)	35.6 (6.1)
SOM (g cm^−2^)	2.6 (0.1)	2.7 (0.5)	2.3 (0.1)	2.2 (0.1)
SOM C (%)	24.3 (3.0)	28.0 (1.9)	24.4 (3.6)	24.4 (1.8)
SOM N (%)	1.1 (0.1)	1.3 (0.1)	1.2 (0.1)	1.2 (0.1)
SOM C/N ratio	21.4 (0.3)	22.0 (0.1)	21.1 (0.3)	20.6 (0.2)
pH	6.0 (0.1)^a^	6.2 (0.04)^ab^	6.4 (0.1)^b^	6.2 (0.1)^ab^
Competitiveness (Hegyi)	3.9 (5.4)^a^	1.8 (0.2)^a^	1.8 (0.2)^ab^	4.6 (3.1)^b^

HP, healthy pine; DFP, defoliated pine; DP, dead pine; H-O, Holm-oaks; SOM, soil organic matter.

In brackets, standard error of the mean. Different letters indicate significant differences between microhabitats at the 0.05 significant level (one-way ANOVA).

## Results

### Chemical composition of SOM related to vegetation and tree health

We did not find any significant differences in SOM composition among the four different area of influence as observed in the ^13^C CP NMR spectra, including the three different indexes under study (Aliphatic C, recalcitrancy, and aromaticity; Table 1 and Table S1). Similarly, no significant differences between treatments could be extracted from the Turkey′s post hoc test (data not shown). In terms of tendencies associated with tree die-off (defoliation and mortality) and chemical composition of SOM, we could not observe any significant (*P*-value <0.05) correlation between degree of defoliation and SOM quality and/or quantity (Table S2). Tree mortality was associated with significantly higher values of soil respiration and pH with respect to the soils under HPs (Table 2). We did not find any significant differences among microbiomes in soil temperature, but soil moisture was statistically higher under DFPs than under the rest of canopy types (Table 2). The Hegyi competition index was significantly higher for HOs with respect to pines, whereas no significant differences were found between healthy, defoliated, and DPs (Table 2). Chemical characteristics obtained from the Catalan Ecological and Forestry Inventory (IEFC; http://www.creaf.uab.es/iefc/) indicates small differences between pine needle and HO leaf chemistry (Table 3). These differences were mainly due to lower values of Calcium (Ca) and Potassium (K) and slightly higher values of Carbon (C) in pines than in HOs. The C/N ratios were thus lower in HO leaves than in pine needles, suggesting a higher palatability of HO leaf litter.

**Table 3:** Chemical composition (mean and standard deviation in brackets) of leaves of the two studied species, according to the data obtained from the Ecological and Forestry Catalan inventory for the studied location

g per 100 g	*Pinus sylvestris*	*Quercus ilex*
C	51.3 (3.1)	49.6 (1.5)
N	1.3 (0.2)	1.4 (0.25)
P	0.11 (0.03)	0.09 (0.03)
S	0.12 (0.05)	0.13 (0.05)
Ca	0.62 (0.21)	0.92 (0.29)
Mg	0.14 (0.04)	0.15 (0.06)
K	0.48 (0.21)	0.54 (0.18)

### Bacterial overall diversity and taxonomic composition

**Figure 3:**
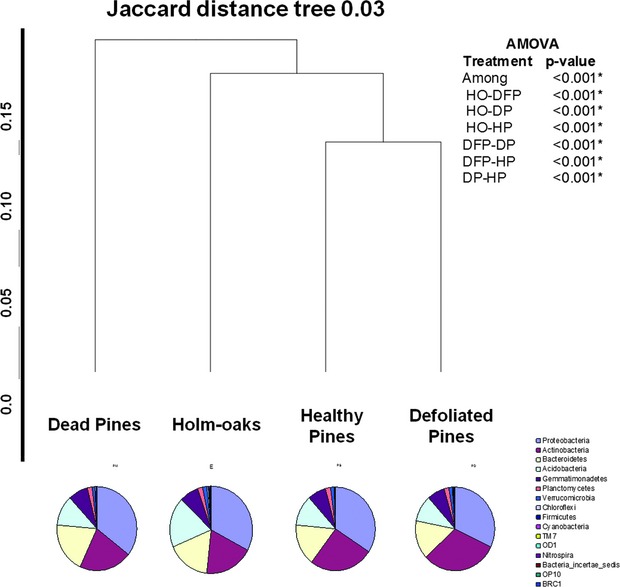
Cladogram of the four microbioma rizospheres based on Jaccard distance**.** Pie charts below each branch represent the relative abundance of the different phyla. Values of the analysis of molecular variance performed in pair-wise comparisons are provided on the right corner. See Table 1 for abbreviations.

Pyrosequencing-based analyses and subsequent statistical inference produced from 11,088–22,000 prokaryotic sequences per treatment (Table S3). Sequences clustered in OTUs on the basis of a 3% taxonomic distance yielded values above 2000 OTUs (Table S3, Fig. S1). The Chao index values show a richness of 3691 different OTUs, whereas the Shannon–Weaver index (H) yielded diversity values of up to 7 (trimmed results obtained from the area of influence of DPs, see Table S3). The distribution of the different taxa is represented in Figure 3. Proteobacteria was the most represented phylum in all the samples (30% of the total bacterial population on average), followed by Actinobacteria, Bacteroidetes, and Acidobacteria. Those four phyla comprised 80% of the total bacterial population. Gemmatidomonas Planctomycetes, Verrucomicrobia, Cloroflexibacterias, and Firmicutes were less well represented.

### Differences in Bacterial community related to vegetation and tree health

There were substantial differences in richness and diversity between the four microbial rhizospheres (Table S3). The Shannon–Weaver index indicates that soil bacterial diversity was higher under DPs and HOs than under healthy and DFPs. Bacterial community in the area of influence of oaks was also richer than in the other microhabitats, according to the Chao index, whereas DFPs showed the lowest richness.

At the OTU level (3% dissimilarity), the AMOVA concluded that all four bacterial communities were different from each other at the 0.001 level of significance (right panel Fig. 3). The differences were maintained in pair-wise comparisons (between treatments). The analyses of similarity with the abundance statistics tool used to calculate Jaccard distance indicates that the bacterial community under DPs was distinct from the communities under living vegetation, whether Healthy or DFPs or Holm-oaks (Fig. 3). Also, healthy and DFPs formed a single cluster, indicating a strong similarity in their bacterial communities. The dissimilarity observed in the bacterial community under DPs were maintained across different taxonomic levels, that is, a gradient of dissimilarity from 0% to 20% (Fig. S2), and this was further supported by the typically lower Jaccard coefficients (1-Jaccard distance) obtained when comparing the dead-pine bacterial communities with the others (Fig. S3).

**Figure 4:**
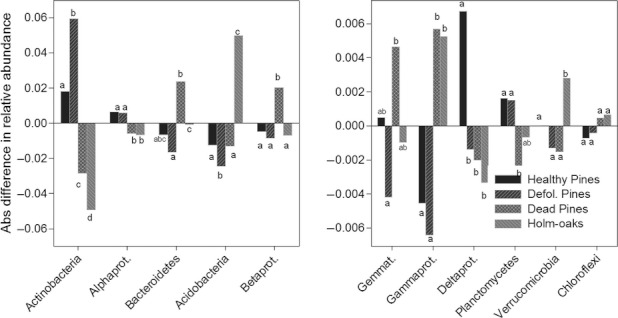
Differences in the relative abundance of the most representative soil bacterial phyla/classes (Proteobacteria is presented at the class level) in relation to the respective average in relative abundance among the four sampled microhabitats (three pine dieback stages and holm-oak). Taxa were arranged in panels according to their relative abundance, that is, Actinobacteria being on average the most abundant and Chloroflexibacteria on average the less abundant of the reported taxa. Different letters indicate significant differences between microhabitats at the 0.001 *P*-level using the approach reported in Cole et al. (2007, 2009). Alphaprot. = AlphaProteobacteria; Betaprot. = BetaProteobacteria; Gemmat. = Gemmatimonas; Gammaprot. = GammaProteobacteria; Deltaprot. = DeltaProteobacteria; Chloroflexi = Chloroflexibacteria.

**Figure 5:**
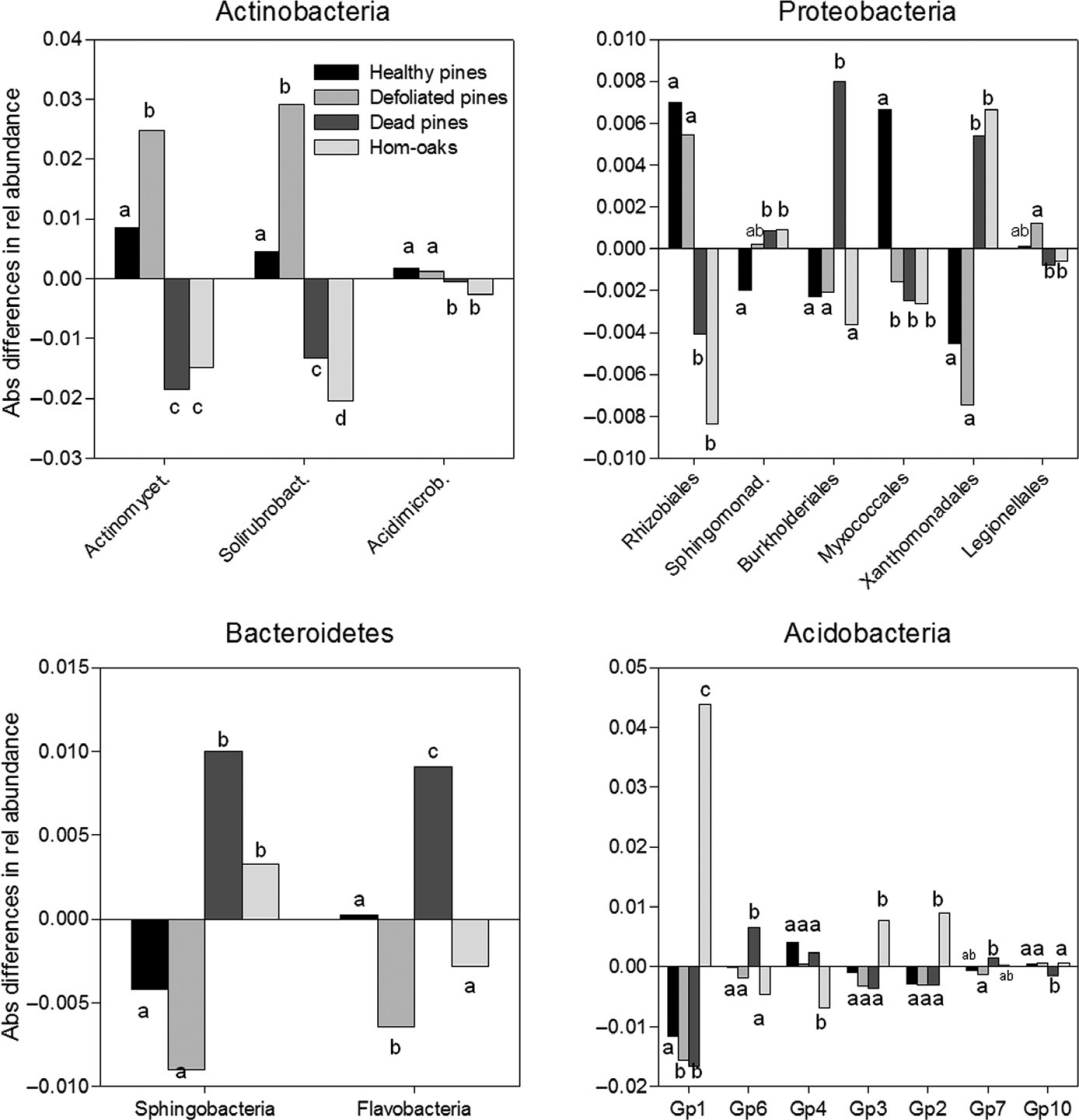
Differences in the relative abundance of the most representative soil bacterial orders in relation to the respective average relative abundance among the four sampled microhabitats (three pine dieback stages and holm oak). Taxa were arranged in panels according to their relative abundance. Different letters indicate significant differences between microhabitats at the 0.001 *P*-level using the approach reported in Cole et al. (2007, 2009). Solirubrobact = Solirubrobacterias; Acidimicrob = Acidimicrobia; Sphingomonad = Sphingomonadales.

There were significant differences in the bacterial community composition associated with plant species and the degree of defoliation or death (Figs. 3–5, Table 4). At the phyla/class level (Fig. 4, Table 4), the relative abundance of several groups (Alphaprotobacteria, Gammaprotobacteria, and Deltaprotobacteria) in pine microhabitats increases along the die-off gradient (form Healthy to Defoliated and Dead trees) converging with the values found in the HO microhabitat. This convergent trend was not observed in other cases, such as Acidobacteria, where the abundance in HOs habitats was always higher than in pine habitats.

**Table 4:** Number of taxa (genus) that comprise each of the phylum/class/orders of Figures 3 and 4

Phylum/class	HO	HP	DFP	DP	Order	HO	HP	DFP	DP
Actinobacteria	32	24	30	44	Actinomycetales	37	38	37	54
					Acidimicrobiales	2	1	3	3
					Solirubrobacterales	2	2	2	2
α-Proteobacteria	31	31	28	40	Myxococcales	5	5	7	8
					Sphingomonadales	3	4	4	6
Bacteroidetes	17	16	12	22	Sphingobacteriales	15	15	17	26
					Flavobacteriales	3	2	2	4
β-Proteobacteria	16	15	15	21	Burkholderiales	15	14	15	20
					Rhizobiales	21	22	21	26
Acidobacteria	12	10	11	13					
Gemmatimonadetes	1	1	1	1					
γ-Proteobacteria	13	9	10	15	Legionellales	2	2	2	2
					Xanthomonadales	11	9	9	13
δ-Proteobacteria	4	5	5	7					
Planctomyces	6	6	7	7					
Verrucomicrobia	3	3	3	3					
Chloroflexibacterias	3	3	2	3					

HP, healthy pine; DFP, defoliated pine; DP, dead pine; H-O, Holm-oaks.

Tree decline triggered important changes in soil bacterial communities, as reflected in their taxonomical composition and diversity. With respect to HPs, pine defoliation coincided with a significant increase in the abundance of OTUs species belonging to the Actinobacteria phylum (orders Actynomecetes and Solirubrobacterias) and a decrease in δ Proteobacteria Flavobacteria (Bacteroidetes phylum) and some Acidobacteria orders (Figs. 3–5). Tree mortality was associated with the appearance of 53 new OTUs (30 of them singletons) not present under living vegetation, mainly from the Actinobacteria, Proteobacteria (α and β), and Bacteroidetes phyla, and the appearance of a new bacteria phylum (Deinnoccus-Thermus; Table S4). The area of influence of DPs was, in general, more similar to that observed in the area of influence of HOs than in the area of influence of HPs (Figs. 3–5). At the level of phylum, Actinobacteria was better represented in the area of influence of HPs than in the area of influence of DPs, but better represented than in that of oaks (Fig. 3). The area of influence of DPs was more abundant in β-Proteobacteria, Gemmatimonas, and Verrucomicrobia than the rest of area of influence (Fig. 4) and similar to the HO area of influence in the relative abundance of α- δ- and γ- Proteobacteria and Chloroflexibacterias (Fig. 4). At lower taxonomic levels, the area of influence of DPs also showed strong similarities with that of HOs, as regards the relative abundance of some orders of Actinobacteria (Actinomycetes and Acidimicrobia), Proteobacteria (Rhizobiales, Sphingomonadales, Myxococcxales, Xanthomonadales and Legionellales), and Bacteroidetes (Sphingobacterias; (Fig. 5). However, the relative abundance of most Acidobacteria orders in DPs was more similar to that of healthy and defoliated ones (Fig. 5).

## Discussion

### Changes in SOM associated with tree mortality and secondary succession

Our results suggest that the ongoing eco-physiological (tree senescence) and ecological (secondary succession) changes occurring aboveground were hardly reflected in the quantity and the composition of SOM (Tables 1 and 2; Tables S1 and S2). Both the quality and the quantity of SOM were, apparently, not affected by tree defoliation, subsequent mortality and species replacement. As tree health worsens and mortality eventually occurs, and after 10 years of net negative C balances (due to no direct inputs of above and belowground litter and decomposition of existing SOM), we expected to observe a net increase in the relative abundance of less palatable organic compounds, such as polyphenols (aromaticity index), lipids, and other aliphatic compounds (recalcitrance index). As the input of more labile substrates as well as cellulose and hemicelluloses would decrease and eventually stop under dead canopies, we also expected to find a significant increase in the aliphacity index (A/A-O index) under dead trees. For the same reasons, we also expected to find a substantial decrease in SOM under dead trees, as is generally observed when trees are harvested (Nave et al. 2010)). It seems, however, very unlikely that the important changes in soil C supply associated with tree mortality would not have resulted in parallel loss in quality and/or quality of soil C pools. In this context, the mixture of typically low palatability of pine litter (Currie 1999; Silver and Miya 2001) along with the inhibitory effect that summer droughts have on the decomposition of SOM (e.g., Curiel Yuste et al. 2007) could have resulted in relatively slow C losses, suggesting that changes in soil C could be occurring at a very slow pace following tree mortality. Other possibility is that forest compensates the losses of C in these gaps with fresh C. No differences in fine root biomass and fine root C:N ratio (which could be a proxy for fine root activity; Pregitzer et al. 1998; Burton et al. 2002) were found among microbiomes (Table 2) suggesting that the niche left by dead individuals has been occupied by new roots of surrounding living vegetation. While this could imply a more complete use of microhabitats and their resources below dead trees, it also may imply continuous incorporation of fresh carbon (in the form of mycorrhizal hyphae and/or fine roots) that compensates carbon losses via mineralization, that is, the forest living canopy is investing C in those gaps compensating for the losses associated with decomposition. Whatever the mechanisms involved, the steady state of C stocks in response to different episodes of tree mortality, being the last one a decade ago, indicates that soil C pools were not very sensitive to this environmental perturbation, at least in the time-frame tackled by this study.

### Bacterial community structure and functioning associated with tree mortality and secondary succession

The high values of bacterial diversity (Table S3), together with the abundance distribution of taxons, closely resembled results obtained in other ecosystems with the same technique (e.g., Janssen 2006; Roesch et al. 2007; Acosta-Martínez et al. 2008; Will et al. 2010; Torres-Cortés et al. 2012). Bacteria ubiquity, dispersal capabilities, and abundance (Martiny et al. 2006, Finlay 2002) could help minimize community differences on a micro-local scale, but, as hypothesized, even at very small spatial scales of this study, we found differences in the structure and composition of the microbial communities under different canopies (Figs. 3–5). These observed differences clearly contrasted with the lack of differences in soil C pools observed under different canopies, and suggest that in this natural experiment microbial communities were more sensitive to tree die-off and succession than were soil C dynamics.

The observed differences in taxonomic composition and diversity between soils under healthy canopies (HOs vs. healthy Scots pines; see Figs. 4, 5, Table S3) could hardly be associated with litter chemistry, as observed in other studies (Binkley 1995; Strickland et al. 2009; Ushio et al. 2010), because quantity and/or quality of SOM (Tables 1 and 2) and nutrient composition of leaf litter (Table 3), did not differ substantially under those canopies. Other aromatic compounds, such as species-specific phenols (Kuiters 1990; Kraus et al. 2003), which may strongly affect the composition and structure of soil microbial communities (Kuiters 1990; Kraus et al. 2003; Strickland et al. 2009; Ushio et al. 2010), were not significantly different between canopies either (Tables S1 and S2). The observed differences may, therefore, respond to other species-specific plant-soil interactions not accounted for in this study, for example, species-specific volatile organic compounds emitted by litter and roots, which are known to affect the structure, stoichiometry and functioning of soil microbial communities (Ramirez et al. 2009; Asensio et al. 2012). The degree of specificity in the interaction between plants and microbes seems, therefore, a very complex matter that involve eco-physiological and/or ecological mechanisms far from being understood.

Tree dieback and death were associated with important differences in diversity (Table S3), structure (Fig. 3), and taxonomic composition of soil bacterial communities (Figs. 4, 5). Eco-physiological changes associated with tree defoliation were, in turn, associated with rapid shifts in the abundance of key taxons of the bacterial community (Figs. 4, 5). Indeed, the loss of health of Scots pine on this or similar stands has been associated with a significant drop in plant-tissue nonstructural forms of C (Galiano et al. 2011), which might have also affected resource allocation to different tree compartments (Sala et al. 2010; McDowell 2011) and hence to roots and associated soil microbes. Soil bacterial communities under DPs, whose death is dated about a decade ago (HereŞ et al. 2011), showed important differences in the diversity (Table S3), structure (Fig. 3), and relative abundance of key taxa (Figs. 4, 5) with respect to bacterial communities under living canopies (healthy and DFPs as well as HOs). We found a significant increase in bacterial diversity (Table S3) with respect to HPs and a clear divergence in community structure with respect to living pines (healthy and defoliated; Fig. 3). This increase in bacterial diversity (Table S3) and the occurrence of a number of new taxa not observed under living vegetation (Table S4) indicate that tree mortality may have stimulated the gap colonization by new bacterial taxa during the last decade, as seen in the early succession stages of microbial community colonization (Huston 1994; Jackson 2003).

Besides the relative increase observed in diversity, pine mortality-driven changes in key abundant taxa, for example, Actynomycetes, Solirubrobacterias, Rhizobiales, or Xantomonadales, pointed towards a convergence of the bacterial communities under dead trees and HOs (Figs. 3–5, and Figs. S2 and S3). Assuming that root-associated organisms are, to some extent, species-specific (Yeates 1999; De Deyn et al. 2003; Bever et al. 1997) our results may indicate that gaps generated by pines are being colonized not only by new bacterial taxa, but also by roots from healthy HOs and their associated microorganisms. We therefore hypothesize that the observed convergence may respond to an early colonization by new bacterial taxa and new HO roots and microorganisms associated into the space occupied by the dead pine area of influence. As explained above, our results indicates that the niche left by dead individuals has been occupied by new roots of surrounding living vegetation, which given the observed convergence of the bacterial communities may imply a more complete use by healthy HOs of microhabitats and the resources left by DPs. Once a tree is dead, the whole pool organic matter generated becomes very susceptible source of nutrients and energy for exploitation by colonizer plant roots (Sanford 1989; Ostertag 1998).

The Hegyi competition index, on the other hand, does not show that DPs were experiencing a higher demographic pressure than that experienced by healthy or DFPs (Table 2), thus confirming that the colonization of above-ground gaps by HOs was in an early stage with respect to below-ground colonization. Studies of the colonization of forest patches have often focused on above-ground processes, for example, dispersal of plant diasporas or the capacity of the colonizer to establish itself in a new habitat and exploit the resources, including soil water and nutrients (Willson 1992). The colonizer plants may, however, also “import” their own microbial community in order to facilitate their germination and eventual recruitment. Soil microbes are important regulators of plant dynamics and diversity that contribute to plant survival, establishment, and growth, for instance through the fixation and mineralization of nutrients (Landerweert et al. 2001; Van der Heijden et al. 2008). This is mere speculation at present but, nevertheless, additional studies need to be designed to better understand the below-ground mechanisms involved in plant colonization, as well as the above-ground ones.

### Tree die-off and soil ecology: indirect links between taxonomy and ecology of soil bacterial communities

Punctual measurements of soil respiration taken in spring 2010 over the same soils sampled for this study suggest that during this period of the year soil respiration was slightly, but consistently higher under dead individuals with respect to living trees (Table 2). A mixed-effect model approach revealed that variables describing quantity and/or quality of SOM could not explain these differences, but the increase in soil respiration under dead individuals was further stimulated by the presence of HOs in the surroundings (J. Barba, J. Curiel Yuste, J. Martínez-Vilalta, F. Lloret, unpubl. data). While these results indicates a direct relation between drought-induced secondary succession and soil respiration, the observed differences in structure of soil bacterial community under different canopies may help supporting these conclusions. Indeed, we found anomalously high representation of bacteria from the Bacteroidetes and β-Proteobacteria taxa under DPs, where soil respiration was higher (Fig. 3). These are two taxons commonly associated with opportunistic r-strategic bacteria typical of environments rich in labile organic matter (Simon *et al*. 2002, Fazi et al. 2005; Fierer et al. 2007). This finding points to a more copiotrophic, richer in organic matter, environment under DPs with respect to healthy vegetation, which on the other hand contradicts the results obtained with ^13^C CP MAS NMR spectra (Table 1 and Table S1). The higher soil metabolic activity (soil respiration) and the differences in soil microbial ecology (diversity and taxonomic composition) under similar nutritional conditions (same quality, same quantity, and same stoichiometry) points to a more efficient use of resources in the “successional hot-spots” (areas under DPs nearby colonizer HOs) of the forest. Although the lack of replication prevented us from drawing conclusions, our observations adds to a growing number of studies claiming a role of the microbial community in soil C emissions and, in general, in soil C processes (e.g., Balser and Firestone 2005; Waldrop and Firestone 2006a,b; Balser and Wixon 2009; Curiel Yuste et al. 2011; Mackelprang et al. 2011). As at least half of the CO_2_ emitted by terrestrial ecosystems is produced by microbial-mediated decomposition of SOM, understanding climate change-related shifts in the composition and diversity of microbes may be crucial to our understanding of future CO_2_ emissions, particularly from the Mediterranean basin.

## Supporting Information

**Table S1**. Results of ANOVA of the comparison of the different spectra segment values and indexes generated (aromaticity, recalcitrancy, and A/A-O ratio) by the ^13^C NMR analyses.

**Table S2**. Pearson coefficient and P-value of the linear relation between degree of defoliation (1 = Healthy; 2 = Defoliated; 3 = dead) and relative abundance of different spectral fractions of SOM.

**Table S3.** Raw (trimmed) and normalized (at the lowest number of healthy pine) values of different soil bacterial diversity estimators.

**Table S4**. Bacterial taxa observed exclusively under dead pine rhizosphere and its relative abundance (%).

**Figure S1.** Rarefaction curves obtained from the data sets clustered using the default parameters for the RDP Clustering function. HP = healthy pine; DFP = defoliated pine; DP = dead pine; HO = Holm-oak.

**Figure S2.** Cladogram of the four microbioma rizospheres based on Jaccard distance at different levels of dissimilarity (1%, 3%, 5%, 10%, 15%, and 20%). The red circle highlights the dead pine clades.

**Figure S3.** Pair-wise Jaccard coefficients of similarity (1-Jaccard distance) of the four sampled microhabitats. HP = healthy pine; DFP = defoliated pine; DP = dead pine; HO = Holm-oak. Highlighted in gray are the Jaccard coefficients at the 3% (OUT's level) and 5% (genus level) dissimilarities.
