# Supplementary material for: Changes in soil bacterial community triggered by drought-induced gap succession preceded changes in soil C stocks and quality
Source: Ecol Evol. 2012 Nov 2;2(12):3016–31. doi: 10.1002/ece3.409 (PMC3538997; doi:10.1002/ece3.409)

**Suplementary Material**

|  | | Sum of squares | df | Quadratic mean | F | Sig. |
| --- | --- | --- | --- | --- | --- | --- |
| **carbonyl C** | Inter-grupos | 17,113 | 3 | 5,704 | ,291 | ,831 |
|  | Intra-grupos | 215,580 | 11 | 19,598 |  |  |
|  | Total | 232,694 | 14 |  |  |  |
| phenolic C | Inter-grupos | 16,889 | 3 | 5,630 | ,453 | ,720 |
|  | Intra-grupos | 136,651 | 11 | 12,423 |  |  |
|  | Total | 153,540 | 14 |  |  |  |
| aryl-C | Inter-grupos | 26,695 | 3 | 8,898 | ,302 | ,824 |
|  | Intra-grupos | 324,311 | 11 | 29,483 |  |  |
|  | Total | 351,006 | 14 |  |  |  |
| di-O-alkyl | Inter-grupos | 33,757 | 3 | 11,252 | ,562 | ,651 |
|  | Intra-grupos | 220,097 | 11 | 20,009 |  |  |
|  | Total | 253,854 | 14 |  |  |  |
| O-alkyl | Inter-grupos | 255,587 | 3 | 85,196 | ,625 | ,613 |
|  | Intra-grupos | 1498,826 | 11 | 136,257 |  |  |
|  | Total | 1754,414 | 14 |  |  |  |
| Metoxyl C | Inter-grupos | 63,389 | 3 | 21,130 | ,558 | ,654 |
|  | Intra-grupos | 416,592 | 11 | 37,872 |  |  |
|  | Total | 479,981 | 14 |  |  |  |
| Alkyl C | Inter-grupos | 144,546 | 3 | 48,182 | ,404 | ,753 |
|  | Intra-grupos | 1310,450 | 11 | 119,132 |  |  |
|  | Total | 1454,996 | 14 |  |  |  |
| Aromaticity | Inter-grupos | 12,629 | 3 | 4,210 | ,230 | ,873 |
|  | Intra-grupos | 201,011 | 11 | 18,274 |  |  |
|  | Total | 213,640 | 14 |  |  |  |
| recalcitrancy | Inter-grupos | ,009 | 3 | ,003 | 1,197 | ,356 |
|  | Intra-grupos | ,027 | 11 | ,002 |  |  |
|  | Total | ,035 | 14 |  |  |  |
| A/A-O ratio | Inter-grupos | ,017 | 3 | ,006 | ,451 | ,722 |
|  | Intra-grupos | ,136 | 11 | ,012 |  |  |
|  | Total | ,152 | 14 |  |  |  |

**Table S1**. Results of ANOVA of the comparison of the different spectra segment values and indexes generated (Aromaticity, Recalcitrancy and A/A-O ratio) by the ^13^C NMR analyses.

|  | **Pearson coefficient** | **p-value** |
| --- | --- | --- |
| **carbonyl** | -,292 | ,483 |
| **phenolic** | -,296 | ,477 |
| **aryl** | -,342 | ,408 |
| **di-O-alkyl** | -,391 | ,338 |
| **O-alkyl** | -,277 | ,506 |
| **Metoxyl** | -,609 | ,109 |
| **Alkyl** | -,420 | ,301 |
| **Suma** | -,393 | ,336 |
| **Aromaticity** | ,580 | ,132 |
| **recalcitrancy** | ,491 | ,217 |
| **A/A-O ratio** | ,237 | ,483 |
| **%C** | ,235 | ,543 |
| **%N** | ,357 | ,345 |
| **C:N** | -,380 | ,313 |

**Table S2.**

| Sample |  | distance | N | clusters | Chao | LCI95 | UCI95 | H' | varH | E | Coverage |
| --- | --- | --- | --- | --- | --- | --- | --- | --- | --- | --- | --- |
| HP | Trimmed | 0.03 | 11088 | 2161 | 3576 | 3353 | 3841 | 6.7 | 0.00021 | 0.87215 | 91.108 |
|  | Normalized | 0.03 | 11088 | 2161 | 3577 | 3354 | 3841 | 6.70 | 0.00021 | 0.87215 |  |
| DFP | Trimmed | 0.03 | 13635 | 2417 | 3725 | 3525 | 3962 | 6.8 | 0.00017 | 0.87361 | 92.549 |
|  | Normalized | 0.03 | 11088 | 2151 | 3281 | 3101 | 3495 | 6.73 | 0.0002 | 0.87735 |  |
| DP | Trimmed | 0.03 | 22911 | 3380 | 5166 | 4926 | 5442 | 7.03 | 0.00011 | 0.86514 | 94.038 |
|  | Normalized | 0.03 | 11088 | 2279 | 3530 | 3337 | 3759 | 6.85 | 0.00019 | 0.88582 |  |
| H-O | Trimmed | 0.03 | 13495 | 2567 | 3980 | 3771 | 4225 | 6.91 | 0.00016 | 0.88004 | 91.827 |
|  | Normalized | 0.03 | 11088 | 2309 | 3692 | 3480 | 3942 | 6.85 | 0.0002 | 0.88478 |  |

**Table S3.** Raw (Trimmed) and normalized (at the lowest number of Healthy Pine) values of different soil bacterial diversity estimators. HP: Healthy Pine; DFP: Defoliated pine; DP: Dead Pine; HO: Holm-oaks. N = number of sequences; Clusters = total number of OTUs at 0.03 distance; Chao index of richness; LCI95 and UCI95: Lower and upper Confidence intervals of Chao index at the 0.05 probability, respectively; H’ = Shannon-Weaver diversity index ; varH= variance of H’; E = Evenness considering H’. Different letters in H’ indicate significant differences between microbial areas of influence.

| *phylum* | *order* | *family* | *genus* | *Relative abundance* |
| --- | --- | --- | --- | --- |
| Actinobacteria | Actinomycetales | Actinosynnemataceae | Saccharothrix | 0,0262 |
| Actinobacteria | Actinomycetales | Actinosynnemataceae | Umezawaea | 0,0087 |
| Actinobacteria | Actinomycetales | Bogoriellaceae | Georgenia | 0,0044 |
| Actinobacteria | Actinomycetales | Micrococcaceae | Nesterenkonia | 0,0044 |
| Actinobacteria | Actinomycetales | Micromonosporaceae | Micromonospora | 0,0175 |
| Actinobacteria | Actinomycetales | Micromonosporaceae | Catellatospora | 0,0044 |
| Actinobacteria | Actinomycetales | Nocardiopsaceae | Thermobifida | 0,0044 |
| Actinobacteria | Actinomycetales | Promicromonosporaceae | Promicromonospora | 0,0087 |
| Actinobacteria | Actinomycetales | Propionibacteriaceae | Propionibacterium | 0,0044 |
| Actinobacteria | Actinomycetales | Pseudonocardiaceae | Saccharomonospora | 0,0087 |
| Actinobacteria | Actinomycetales | Pseudonocardiaceae | Saccharopolyspora | 0,0087 |
| Actinobacteria | Actinomycetales | Pseudonocardiaceae | Allokutzneria | 0,0044 |
| Actinobacteria | Actinomycetales | Pseudonocardiaceae | Crossiella | 0,0044 |
| Actinobacteria | Actinomycetales | Pseudonocardiaceae | Thermocrispum | 0,0044 |
| Actinobacteria | Actinomycetales | Streptosporangiaceae | Planotetraspora | 0,0044 |
| Actinobacteria | Actinomycetales | Thermomonosporaceae | Thermomonospora | 0,0087 |
| Actinobacteria | Actinomycetales | Thermomonosporaceae | Actinocorallia | 0,0044 |
| Proteobacteria | Alteromonadales |  |  | 0,0044 |
| Proteobacteria | Burkholderiales | Alcaligenaceae | Bordetella | 0,0219 |
| Proteobacteria | Burkholderiales | Alcaligenaceae | Pusillimonas | 0,0087 |
| Proteobacteria | Burkholderiales | Comamonadaceae | Schlegelella | 0,0175 |
| Proteobacteria | Burkholderiales | Comamonadaceae | Ramlibacter | 0,0087 |
| Proteobacteria | Burkholderiales | Oxalobacteraceae | Janthinobacterium | 0,0131 |
| Proteobacteria | Chromatiales | Chromatiaceae | Rheinheimera | 0,0044 |
| Proteobacteria | Myxococcales | Kofleriaceae | Kofleria | 0,0044 |
| Proteobacteria | Myxococcales | Nannocystaceae | Enhygromyxa | 0,0044 |
| Proteobacteria | Pseudomonadales | Moraxellaceae | Acinetobacter | 0,0044 |
| Proteobacteria | Pseudomonadales | Pseudomonadaceae | Azotobacter | 0,0044 |
| Proteobacteria | Rhizobiales | Brucellaceae | Ochrobactrum | 0,0087 |
| Proteobacteria | Rhizobiales | Hyphomicrobiaceae | Filomicrobium | 0,0044 |
| Proteobacteria | Rhizobiales | Rhizobiaceae | Ensifer | 0,0087 |
| Proteobacteria | Rhodocyclales | Rhodocyclaceae | Azoarcus | 0,0044 |
| Proteobacteria | Rhodocyclales | Rhodocyclaceae | Denitratisoma | 0,0044 |
| Proteobacteria | Rhodospirillales | Acetobacteraceae | Craurococcus | 0,0044 |
| Proteobacteria | Rhodospirillales | Rhodospirillaceae | Skermanella | 0,0787 |
| Proteobacteria | Sphingomonadales | Erythrobacteraceae | Erythrobacter | 0,0087 |
| Proteobacteria | Sphingomonadales | Sphingomonadaceae | Sphingopyxis | 0,0219 |
| Proteobacteria | Xanthomonadales | Sinobacteraceae | Alkanibacter | 0,0044 |
| Bacteroidetes | Bacteroidales | Marinilabiaceae | Alkaliflexus | 0,0219 |
| Bacteroidetes | Flavobacteriales | Flavobacteriaceae | Gelidibacter | 0,0044 |
| Bacteroidetes | Sphingobacteriales | Cyclobacteriaceae | Algoriphagus | 0,0131 |
| Bacteroidetes | Sphingobacteriales | Cyclobacteriaceae | Aquiflexum | 0,0044 |
| Bacteroidetes | Sphingobacteriales | Cyclobacteriaceae | Aureispira | 0,0044 |
| Bacteroidetes | Sphingobacteriales | Cyclobacteriaceae | Lewinella | 0,0044 |
| Bacteroidetes | Sphingobacteriales | Cytophagaceae | Pontibacter | 0,0087 |
| Bacteroidetes | Sphingobacteriales | Cytophagaceae | Sporocytophaga | 0,0044 |
| Bacteroidetes | Sphingobacteriales | Flammeovirgaceae |  | 0,0044 |
| Bacteroidetes | Sphingobacteriales | Rhodothermaceae | Salinibacter | 0,0044 |
| Bacteroidetes | Sphingobacteriales | Sphingobacteriaceae | Parapedobacter | 0,0131 |
| Firmicutes | Bacillales | Paenibacillaceae | Brevibacillus | 0,0044 |
| Firmicutes | Bacillales | Paenibacillaceae | Thermobacillus | 0,0044 |
| Firmicutes | Bacillales | Staphylococcaceae | Jeotgalicoccus | 0,0044 |
| Firmicutes | Clostridiales | Gracilibacteraceae | Lutispora | 0,0044 |
| Firmicutes | Clostridiales | Peptococcaceae | Desulfotomaculum | 0,0087 |
| Firmicutes | Clostridiales | Ruminococcaceae |  | 0,0044 |
| Verrucomicrobia | Puniceicoccales | Puniceicoccaceae |  | 0,0087 |
| Verrucomicrobia | Verrucomicrobiales | Verrucomicrobiaceae | Prosthecobacter | 0,0044 |
| Deinococcus-Thermus | Deinococcales | Trueperaceae | Truepera | 0,0175 |

**Table S4**. Bacterial taxa observed exclusively under dead pine rhizosphere and its relative abundance (%).

**Figure captions**

**Supplementary Figure 1.** Rarefaction curves obtained from the data sets clustered using the default parameters for the RDP Clustering function. HP= healthy pine; DFP=Defoliated pine; DP= Dead pine; HO= Holm-oak.

**Supplementary Figure 2.** Cladogram of the four microbioma rizospheres based on Jaccard distance at different levels of dissimilarity (1%, 3%, 5%, 10%, 15% and 20%). The red circle highlights the dead pine clades.

**Supplementary Figure 3.** Pair wise Jaccard coefficients of similarity (1-Jaccard distance) of the four sampled microhabitats (HP= healthy pine; DFP=Defoliated pine; DP= Dead pine; HO= Holm-oak). Highlighted in grey are the Jaccard coefficients at the 3% (OUT´s level) and 5% (genus level) dissimilarities.

**Supplementary Figure 1**


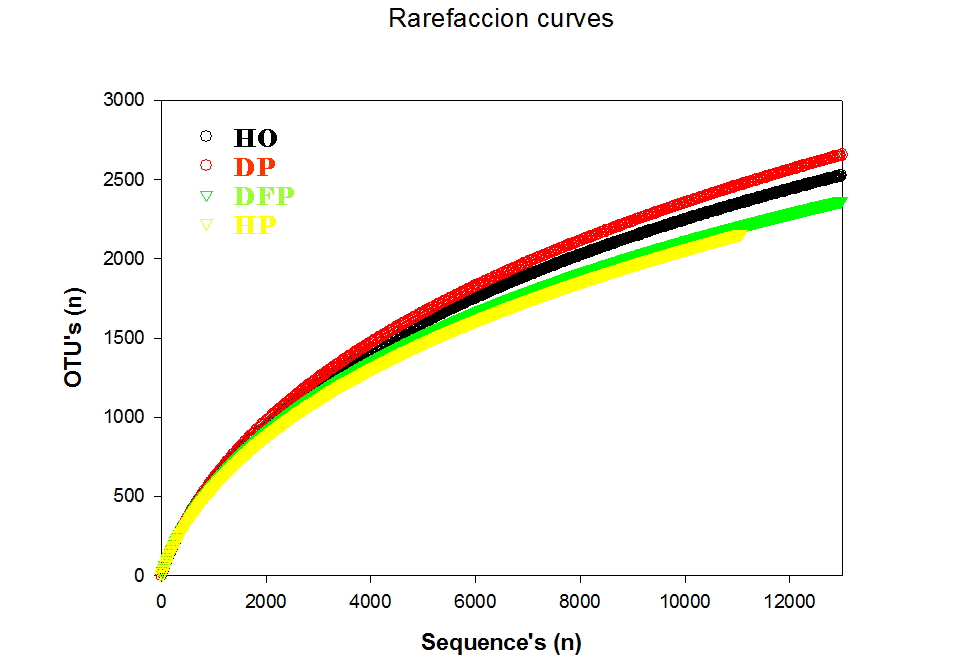


**Supplementary Figure 2**


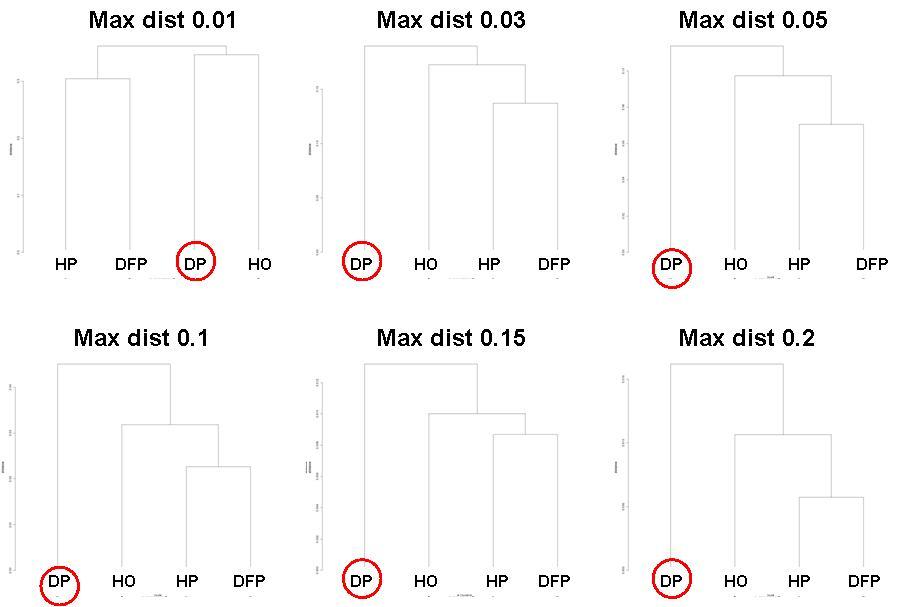


**Supplementary Figure 3**


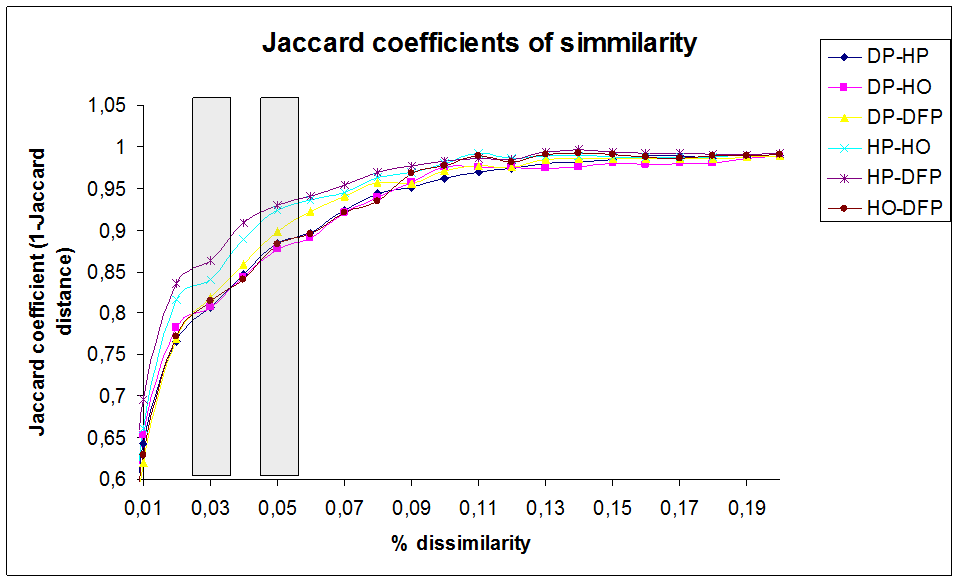

Supplement: Supplementary file 1 [file ece30002-3016-SD1.docx]
